# Anti-Mercury and Anti-Mineral Doctors

**Published:** 1848

**Authors:** 


### ARTICLE IV.

### *Anti-Mercury and Anti-Mineral Doctors.*

Within a few years, there has appeared among the American people a hybrid or mongrel race of physicians—or of men who aspire to the honorable term—whose main reliance for success in business consists in the repudiation of the mineral preparations of the Materia Medica. As far as they possess any correct notions of medical science, these anti-mineral doctors owe all their enlightenment to the standard works of the regular profession. It is on the sole ground of the resurrection of an entombed prejudice that they fix their hopes of notoriety and of patronage from society. The ancient idea of the Galenicans, or followers of Galen, that remedies obtained from the earth were in all cases injurious to the human system, and that vegetable substances alone were proper to restore men to health when afflicted by disease, has been drawn out of its dark and putrid resting place, and made the object of a fond idolatry by these *herb and root doctors.* And by puffing and paint, fustian and foolery, they have so dilated the form and discolored the features of their idol, as to render it difficult of recognition. But after all this lavish pains-taking to create a *living something* out of a *dead nothing*, there remains but one consolation for them, which is this, that their credulous stolidity has mistaken a dead lion's skin for a living dog. Solomon said, and perhaps they remember this—if, indeed, they ever have troubled their weak heads with what Solomon has written—that a living dog is better than a dead lion. Now, the dog can bark, and, if you let him, may bite you; but a dead lion can neither threaten you by his menacing presence, nor lacerate your flesh by his onset.

By what characteristic are these anti-mineral doctors distinguished? We answer, by six very noticeable attributes of character.

*First.*—By an obvious defect of a sound elementary education;

*Second.*—By their illogical mode of reaching their distinctive conclusions;

*Third.*—By their vituperation of the regular profession;

*Fourth.*—By their appeals to popular prejudice;

*Fifth.*—By their quick and spontaneous amalgamation with every current tom-foolery of the day; and

*Sixth.*—By the limited range of their favorite scheme of practice.

Perhaps there is no one point connected with the general experience of mankind more patent than that of exclusiveness and intolerance attaching themselves to restricted mental culture. Ignorance is the mother of bigotry; a mind unblessed by the genial light of a liberal education is always a mind rigid and sterile, incapable of enlarged views, and ever fixed in its narrow conceptions. One of the most significant fruits of a liberal education is the flexibility acquired by the mind for a ready adaptation to the reception of truth, in every varied phase in which it may be offered. It should be a matter of no surprise to a philosophical observer to find that these men, who are denominated botanical practitioners of medicine, are of crude mental culture; that their primary education is exceedingly restricted; and consequently that their faculties are undisciplined, their conceptions low and puerile, while their vanity is inordinate, with a correspondent contempt of the intellect of those who do not symbolize with them in their peculiarities of thought.

As a resulting phenomenon of defective ednoation, these "root and herb doctors" display a wonderful innocence in matters of logic. They seem incapable of making two propositions cohere, or of drawing a correct inference from well established premises. Thus, in despite of anatomy, of physiology, and of pathology, they strenuously contend that no mineral substance must be administered to a sick person. Anatomy disproves this position. The boney structure of man is essentially mineral, as far as the strength of the bones is concerned. Phosphate of lime is the earthy material of oseous formation —but phosphate of lime is composed of phosphoric acid and lime: lime is composed of oxygen and calcium. Calcium is metallic; consequently the bones contain a metal as the basis of their composition. Again, the blood of man contains various salts, such as the chloride of sodium and potassium, carbonate of soda, phosphate of lime, soda and magnesia, sulphate of soda and per-oxide of iron. Deprive the blood of these mineral substances and the animal perishes; diminish the normal quantity of these materials and you have disease. Therefore, as in some cases of disease there does exist a comparative destitution of earthy matters, and especially of iron in the blood, enlightened reason dictates the administration of such mineral substances as may supply, through the vital processes, such absence of the healthy ingredients of the circulating fluid.

The brain of man contains phosphorus, soda and iron, in minute quantities. Is it wrong, in certain nervous affections, to supply nature with these substances, which, if needs be, may be aidant of her want?

And what does physiology teach us in reference to this point? Take, for instance, the ingredients which enter into the dietetic support of man, and do we not find common salt, chloride of sodium, an indispensible article, deprived of which the digestive and assimilative organs utterly fail in the performance of their functions? And this mineral substance enters the human blood, and there plays an important part in its physiological relations. Iron is always a part of healthy blood, and is constantly entering the circulation through our aqueous and solid aliments.

What is water, in its purest state? Certainly not a vegetable production: it resembles inorganic or mineral substances, far more than vegetable. But man never partakes of perfectly unmixed water: it is always impregnated with earthy or atmospheric ingredients, whether taken in the form of spring, well, hydrant, or cistern water.

And the atmosphere, what is it? Not a vegetable, but a fluid, composed of oxygen and nitrogen, with a small portion of carbonic acid gas. It resembles mineral bodies nearer than it does vegetable.

Pathology teaches that diseases is an unnatural event, and not a mere excess of physiological action. The idle disputation indulged in by some speculators, respecting the final cause of disease, can never conduct to a true view of its proximate character. Thus, to aver that fever is but an effort of nature to get rid of some peccant material, is a daring violation of all just philosophy. Why should the physician talk thus, when his direct relation to disease lies in an entirely different direction than that of such transcendentalism? He is called in, not to tell his patient that disease is blessing, that nature is dealing very beneficially with him in the pains and agitations of his frame, and that she must be let alone in her gentle inflictions. This is as good philosophy as to say that a mob in civil society is a good thing, for it is nothing more than the vis popularis reacting against an evil. Or that the *populi fœx*, like the peccant matter in the blood, needs a discharge, and that in the one case we have a fever, in the other a mob: one an insurrection against the laws of the body politic, the other against the laws of the body corporal.

Disease is an evil, a violation of the laws of nature, a departure from the physiological processes of the animal economy, and must be met by foreign influences—by agents not natural but medicinal; not hygienic, but therapeutical.

These agents, whether obtained from animals, as cantharides and musk, or from vegetables, as quinine, ipecac, opium, &c., or from minerals, as iron, salt, mercury, antimony, &c., are all endowed, as far as they possess curative powers, with an energy in virtue of which they arrest disease and restore health. The activity and permanent effects of a medicine do not depend upon its mere mineral, animal, or vegetable, but upon its inherent properties, and on its capacity for combination. Thus opium as derived from the poppy is inherently active in the production of narcotic effects. But its curative properties are increased by detaching, from its combination with the other elements, morphine, and combining this active element with a mineral acid, such as sulphuric, or muriatic.

Do we cure disease by water? This is not a botanical remedy. To be consistent, these men must abandon water altogether in the treatment of burns and other injuries, and never apply it, in cases of fever, to the surface of the body. But the steam doctors do apply warm water and give warm aqueous drinks, which only proves that they are so little capable of logical reasoning as to contradict their own professions of being exclusive "root and herb doctors."

"Botanicals, steamers, eclectics, root and herb doctors," for by these various appellations are these doctor-errants known in society, however they differ in other respects, yet in this one particular they all agree, namely, in abuse of the regular profession of medicine. They luxuriate and revel in obloquy, and esteem it high merit to traduce with endless vilifications "the mineral doctors," the "mercurial doctors," or, in other words, the regular faculty of physic.

> "Still runs the tongue, in raging vein,
> E'en to the dregs and squeezings of the brain;
> Strain out the last dull droppings of their sense,
> And talk with all the rage of impotence."

The reiterated appeals made by these men to popular prejudice, and their ceaseless efforts to enlist the sympathy of the public in their behalf, explicitly declare that they feel conscious of their inability to rest their pretensions on the permanent basis of science. There is an unobtrusiveness about science utterly at variance with the spirit of gasconading evinced by these irregular practitioners. A man devoted to scientific inquiries, who delights in the disclosures of truth in nature, is apt to cherish such a self-respect as will forbid all idle parade and vaporing assumption.

But the empirical pretender is ever addicted to self adulation: the music of his praise, uttered by his own tongue, is ever vibrating on his ears, and all men may take knowledge of him that he is steeped, up to the lips, in the muddy pool of his own vain glory. Thus immersed in the stagnant waters of his own egotism, the quack is forever prating to all upon whom he can inflict his vain talk, that he is veritably King Log, sent down from Jupiter to reign over all the fenny region about him.

The endless twaddle about mercury in which the "botanicals" indulge, is replete with two precious qualities of mental greatness; a vast conception of their own wisdom, and an utter aversion of any improvement. They remind us of the man who gravely asserted that he would never employ a doctor who so far felt his ignorance as to have recourse to books; for as he assured us, no man is fit to practice medicine who believes that he does not known enough to carry him through life, without learning any more from authors. Or, like the "colored doctor" who gained great notoriety among us several years ago, these "root and herb doctors" feel assured that it is all *a gift*—whether from heaven or the demon Paracelsus, it does not require a diviner to determine.

The "botanicals" have a strong affinity for anything extraordinary. Thus they greedily devour all the wonders of animal magnetism, and finding their "roots and herbs" to fail in curing disease, they readily fall to work on the devices of Mesmer. Hocus pocus comes in as succedaneum to lobelia; for while one stirs up the bile and the bowels, the other etherializes the soul, so as to make it quiet under the tribulation of the body.

Lastly, these "eclectics, botanicals, steamers, and root and herb doctors," have reached the *thule ultima*, the uttermost boundary of medical skill. They cure pleurisy without bleeding, by shaking the chest well with a lobelia vomit; remove the effects of an injury to the head by urging more blood to the brain by the same potent herb, and extirpate a cholic by making the bowels of the patient "untwist themselves" by their delectable "Indian physic."

Is a woman in labor, with rigid os uteri? puke her with lobelia. Is a baby cholicky? be sure to give it the same that was good for the mother to hasten birth. Is there retention in the placenta? do not manipulate in any way, but force the after birth by making the woman strain well in the act of emesis.

The lancet, cupping, leeching, blistering, with mercury, antimony, salts, sulphur, iron, the preparations of lead, zinc and copper, and the mineral acids, are all to be abandoned. At whose command? The driveler replies, at the mighty behest of Magnus Apollo, the sage author of that profound system of physic, denominated Thomsonianism; or of that equally captivating and wise scheme of doctoring men, called Beechism. Mighty sons of Apollo, let all hear thy voice and submit to thy guidance!

> Lo! thy dread empire, Chaos! is restored;
> Light dies before thy uncreating word:
> Thy hand, great Anarch! lets the curtain fall,
> And universal darkness buries all.

H.

—*Western Lancet.*

